# Prevalence of carbapenemase-producing *Klebsiella pneumoniae* at a tertiary care hospital in Kathmandu, Nepal

**DOI:** 10.1186/s41182-021-00368-2

**Published:** 2021-09-26

**Authors:** Susil Pyakurel, Mehraj Ansari, Smriti Kattel, Ganesh Rai, Prasha Shrestha, Kul Raj Rai, Anil Kumar Sah, Shiba Kumar Rai

**Affiliations:** 1 Department of Microbiology, Shi-Gan International College of Science and Technology, Kathmandu, Nepal; 2 Key Laboratory of Fujian-Taiwan Animal Pathogen Biology, College of Animal Sciences, Fuzhou, China; 3 Department of Microbiology, Annapurna Research Centre, Kathmandu, Nepal; 4 Department of Microbiology, Nepal Medical College and Teaching Hospital, Kathmandu, Nepal

**Keywords:** Carbapenemase, MDR, Colistin, MHT, Nepal

## Abstract

**Aim:**

Although carbapenem is the last-resort drug for treating drug-resistant Gram-negative bacterial infections, prevalence of carbapenem-resistant bacteria has substantially increased worldwide owing to irrational use of antibiotics particularly in developing countries like Nepal.  Therefore, this study was aimed to determine the prevalence of carbapenemase-producing *K. pneumoniae* and to detect the carbapenemase genes (blaNDM-2 and blaOXA-48) in at a tertiary care hospital in Nepal.

**Materials and methods:**

A hospital-based cross-sectional study was carried out from June 2018 to January 2019 at the Microbiology Laboratory of Annapurna Neurological Institute and Allied Sciences, Kathmandu, Nepal. Different clinical samples were collected and cultured in appropriate growth media. Biochemical tests were performed for the identification of *K. pneumoniae*. Antibiotic susceptibility testing (AST) was performed by the Kirby–Bauer disc diffusion method. The modified Hodge test (MHT) was performed to detect carbapenemase producers. The plasmid was extracted by the modified alkaline hydrolysis method. Carbapenemase-producing *K. pneumoniae* were further confirmed by detecting blaNDM-2 and blaOXA-48 genes by PCR using specific forward and reverse primers followed by gel electrophoresis.

**Results:**

Out of the total 720 samples, 38.9% (280/720) were culture positive. *K. pneumoniae* was the most predominant isolate 31.4% (88/280). Of 88 K*. pneumoniae* isolates, 56.8% (50/88) were multi-drug resistant (MDR), and 51.1% (45/88) were MHT positive. Colistin showed the highest sensitivity (100%; 88/88), followed by tigecycline (86.4%; 76/88). blaNDM-2 and blaOXA-48 genes were detected in 24.4% (11/45) and 15.5% (7/45) of carbapenemase-producing *K. pneumoniae* isolates, respectively*.*

**Conclusion:**

The rate of MDR and carbapenemase production was high in the *K. pneumoniae* isolates. Colistin and tigecycline could be the drug of choice for the empirical treatments of MDR and carbapenemase-producing *K. pneumoniae*. Our study provides a better understanding of antibiotic resistance threat and enables physicians to select the most appropriate antibiotics.

**Supplementary Information:**

The online version contains supplementary material available at 10.1186/s41182-021-00368-2.

## Introduction

*Klebsiella pneumoniae* is an opportunistic Gram-negative bacterium responsible for several of nosocomial infections including urinary tract infections, pneumonia, and septicemia [1]. It is one of the most frequently isolated organisms after *Escherichia coli* from clinical specimens in Nepal [2] and has a greater clinical significance. Irrational and widespread use of antimicrobial agents has led to the increase in antimicrobial resistance in these isolates [3] and the multidrug-resistant (MDR) *K. pneumoniae* isolates have been reported by many workers in Nepal [2, 4, 5]. Such multidrug-resistant *K. pneumoniae* isolates show a high resistance to a broad spectrum of drugs including beta-lactam antibiotics, fluoroquinolones, and aminoglycosides [6]. Until now, carbapenems were considered as the most effective drug against the MDR *K. pneumoniae* and frequently used as the drugs of last resort [7]. However, the overdependence on carbapenems has led to an undesirable increase in the carbapenem-resistant isolates [8].

Carbapenem resistance is mediated by the production of carbapenemases; β-lactamases with versatile hydrolytic capacities or by the combination of outer membrane porin expression disruption [1]. Different carbapenemase genes are responsible for the production of carbapenemases, the most clinically significant carbapenemases are class A (KPC type), class B metallo-β-lactamases (MBLs), i.e., VIM, IMP, and NDM types, and class D carbapenem-hydrolyzing β-lactamases (OXA-48-like enzymes) [9, 10]. The New Delhi metallo-β-lactamases (NDM), first reported in India [11] and OXA-48, first reported in Turkey [12] and both have been reported in *K. pneumoniae*. Since their first detection, both of the carbapenemase genes have spread across the globe [11–14]. Frequent isolation of the carbapenemase-producing isolates poses a significant complication in the treatment. Furthermore, these genes have the potential for dissemination via mobile genetic elements such as plasmids and transposons [15] which increases the risk of widespread dissemination in hospital settings as well as in the community.

In Nepal, the prevalence of carbapenemase-producing *K. pneumoniae* have been reported by several workers [16, 17]; however, data obtained by most of previous studies were mainly based on phenotypic characterization of carbapenemase-producing *K. pneumoniae*, but not by molecular methods [16]. Recently, a few authors have reported the prevalence of blaOXA-48 genes in *K. pneumoniae* ranging from nearly 10 to 70% [16–18]. On the other hand, the NDM series of genes of Indian origin could have been easily circulating in Nepal [16, 19] owing to the Indo-Nepal open border facilitating people to move freely between the two countries. In this study, we therefore, wanted to address this pressing need by detecting the blaNDM-2 and blaOXA-48 genes among the carbapenemase-producing *K. pneumoniae* at a tertiary care center in Nepal. This study helps to determine the prevalence of blaOXA-48 blaNDM-2-producing *K. pneumoniae* and to design apt antibiotic prescription in days to come.

## Materials and methods

### Study design

This cross-sectional study was conducted from June 2018 to January 2019 at Annapurna Neurological Institute and Allied Sciences, Kathmandu, Nepal. The hospital is a 200-bed referral hospital located in the middle of Kathmandu. The hospital provides services for patients of the Kathmandu valley and also, patients referred from other hospitals outside the Kathmandu valley. The study population included patients of all ages and genders visiting the both outpatient and inpatient department of the hospital. The patients under any antimicrobial treatments were excluded from the study.

### Sample collection and identification

The samples included were urine, blood, sputum, CSF, pus, tracheal aspirates and catheter tips. Samples were collected by employing standard microbiological protocol [20]. Repeated samples and samples showing the possible signs of contaminations were excluded from this study. Valid specimens were cultured in suitable culture media as per their requirements. Identification of *K. pneumoniae* was made based on colonial morphology, staining reactions, and various biochemical properties [21].

### Antimicrobial susceptibility testing (AST)

The identified *K. pneumoniae* isolates were subjected to an in vitro antimicrobial susceptibility testing by the modified Kirby–Bauer disc diffusion method as recommended by CLSI guidelines [22]. The antibiotic disks used were ampicillin (10 μg), gentamicin (10 μg), ciprofloxacin (5 μg), co-trimoxazole (25 μg), amikacin (30 μg), ceftazidime (30 μg), cefotaxime (30 μg), imipenem (10 μg), meropenem (10 μg), ertapenem (10 μg), tigecycline (15 μg and colistin (10 μg). *K. pneumoniae* which were resistant to at least 3 different classes of antibiotics were considered as multidrug-resistant strains.

### Screening of carbapenemase producers

Imipenem, meropenem, and ertapenem discs were incorporated in the primary AST plate for the screening of the carbapenemase producers. Isolates showing resistance to at least one of the carbapenem discs mentioned were suspected as possible carbapenemase producers and were processed for a further confirmatory test.

### Confirmation of carbapenemase production

Carbapenemase production was confirmed phenotypically by the Modified Hodge Test (MHT). For this, an overnight suspension of *E. coli* (ATCC 25922) adjusted to a turbidity of the 0.5 McFarland standard was inoculated evenly on the surface of the MHA plate containing 70 μg per ml of ZnSO_4_. After drying at room temperature, meropenem was placed at the center of the plate, and then the screening positive isolates were stroked from the edge of the disk to the periphery of the plate and incubated overnight at 37 °C aerobically. The presence of a clover leaf-shaped inhibition zone was considered carbapenemase production. The confirmed isolates were preserved in 25% glycerol and stored at − 20 °C until further confirmation by molecular test.

### Molecular characterization of blaOXA-48- and blaNDM-2-producing K. pneumoniae

DNA (plasmid) was extracted by the modified alkaline hydrolysis method [23]. The DNA extracts were resuspended in Tris–EDTA (10 mM Tris–HCL, 0.10 mM EDTA, pH 8.0) buffer and stored at 4 °C for further analysis. The DNA extracts were amplified by PCR (Proflex, Thermo Fisher, USA) as described by Dallence et al. [24] for blaOXA-48 genes and Kaase et al. [25] for blaNDM-2 genes. The primers used were: OXA-48 F-GCTTGATCGCCCTCGATT, OXA-48 R-GATTTGCTCCGTGGCCGAAA for blaOXA-48 genes and NDM-2 F CACCTCATGTTTGAATTCGCC, NDM-2 R CTCTGTCACATCGAAATCGC manufactured by Macrogen (Korea). For gene amplification, 4 μl plasmid DNA, 12.5 μl master mix manufactured by Solis Biodyne Company, Estonia, 7.5 μl nuclease-free water, and 0.5 μl each of reverse and forward primers were added to make a final mixture volume of 25 μl. The optimized conditions for the thermal cycling process was initial denaturation at 94 °C for 10 min, denaturation at 94 °C for 40 s, annealing at 60 °C for 40 s, and extension at 72 °C for 1 min with a final extension at 72 °C for 7 min at the end of 30 cycles, followed by maintenance at 4 °C for OXA-48 and initial denaturation at 94 °C for 3 min, denaturation at 94 °C for 50 s, annealing at 54 °C for 50 s and extension at 72 °C for 50 s with a final extension at 72 °C for 5 min at the end of 35 cycles, followed by maintenance at 4 °C for NDM-2. Ethanol precipitation method was used to purify the amplified DNA [26].

Electrophoresis of the amplified DNA was performed in 1.5% agarose gel stained with 0.5 µl ethidium bromide. Finally, the gel was visualized under a UV trans-illuminator for photo-documentation. The molecular weight of the amplified product was estimated using a 1000-bp plus DNA ladder (Solis Biodyne, Estonia). The band of 281 bp and 984 bp were considered positive for the blaOXA-48 and blaNDM-2genes, respectively [24, 25].

**Figure:**
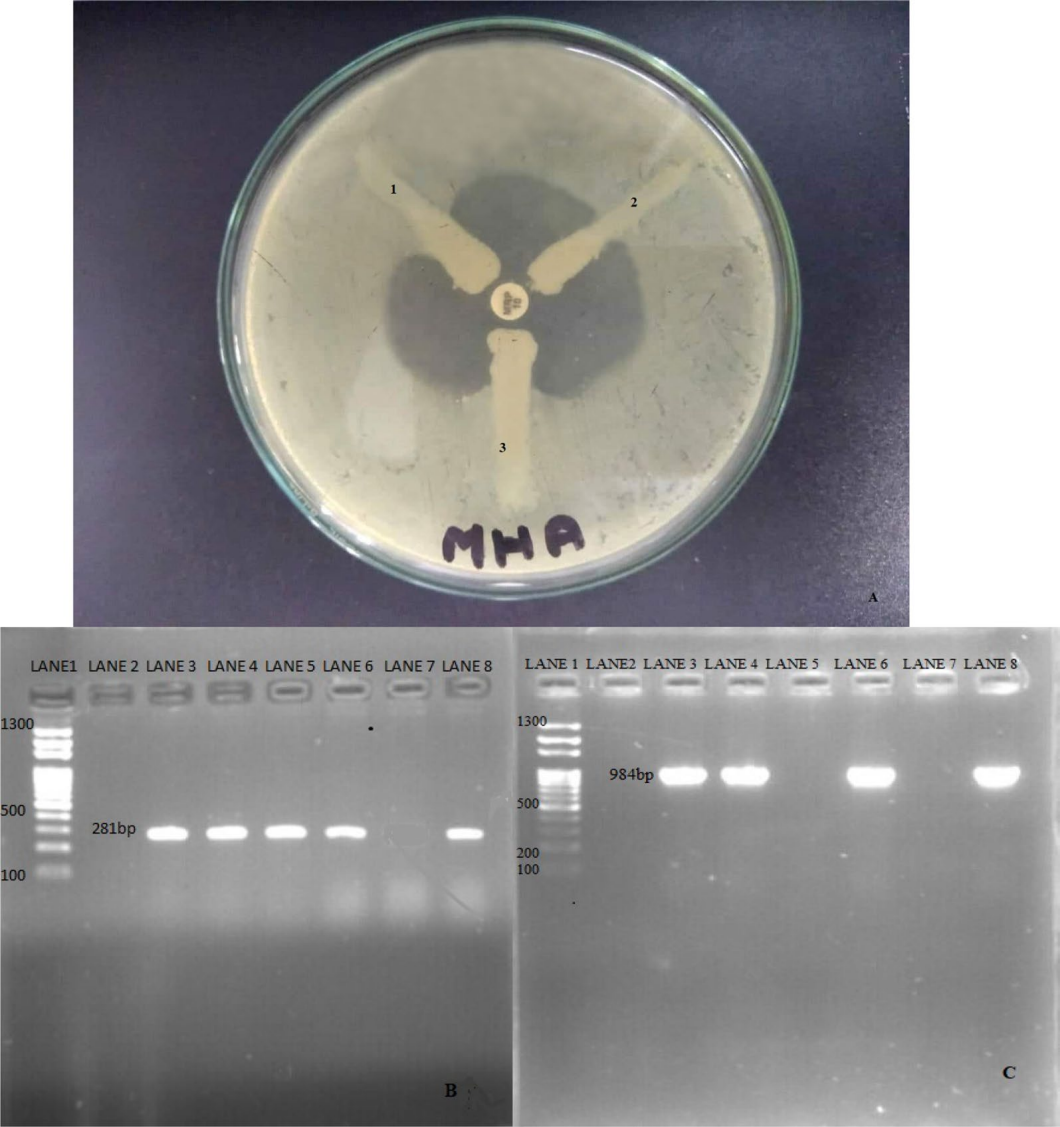
Photograph **A**: Modified Hodge test (MHT): A-negative control, B-positive control and C- test organism (positive). Photograph **B**: Gel electrophoresis of PCR amplicon of blaOXA-48 gene (281 bp) (lane 1 ladder, lane 2 negative control, lane 3 positive control, lanes 4, 5, 6 & 8 positive samples and lane 7 negative sample). Photograph **C**: Gel electrophoresis of PCR amplicon of blaNDM-2 gene (984 bp) (lane 1 ladder, lane 2 negative control, lane 3 positive control, lanes 4, 6 & 8 positive samples and lanes 5 & 7 negative samples)

### Quality control

All the tests were carried out by following standard aseptic techniques and CLSI guidelines [20, 22]. *K. pneumoniae* (ATCC 700603) was used to ensure the performance of newly prepared media and the quality control of AST and MHT. During PCR, quality control was assured by *K. pneumoniae* (ATCC 700603) isolates carrying both the genes under question.

### Statistical analysis

Statistical analysis was performed using Statistical Package for Social Science (SPSS) version 16. Chi-square test at 95% confidence interval (CI) was performed to evaluate the association between demographic variables.

## Results

### Growth positivity

Among the 720 samples collected, 38.9% (280/720) were found culture positive. Of the culture-positive samples, 55.0% (154/280) were Gram-negative, and 45.0% (126/280) were Gram-positive. *K. pneumoniae,* 31.4% (88/280) was the most predominant isolate. Females had a higher isolation rate than males. Tracheal aspirates had the highest 48.3% (42/87) culture positivity followed by sputum 45.7% (58/127), urine 40.4% (110/272), catheter tips 35.9% (32/89), and others. Among the wards, the highest culture positivity was obtained in the ICU 55.1% (98/178), followed by the OPD 42.2% (118/280) and general ward 24.4% (64/262); however, the isolation rate of *K. pneumoniae* was the highest 42.1% (27/64) in the general ward and the least in the ICU (Table 1).

**Table 1:** Growth positivity according to sex, clinical samples, and hospital units

	Growth positive	Gram negative	*K. pneumoniae*	MDR *K. pneumoniae*
	*n* (%)	(%)*	(%)*	(%)**
Sex				
Male (*n* = 392)	144 (36.7%)	68 (47.2%)	37 (25.7%)	23 (62.2%)
Female (*n* = 328)	136 (41.5%)	86 (63.2%)	51 (37.5%)	27 (52.9%)
Sample				
Urine (*n* = 272)	110 (40.4%)	63 (57.2%)	24 (21.8)	11 (45.8%)
Sputum (*n* = 127)	58 (45.7%)	31 (53.4%)	23 (39.7%)	16 (69.6%)
Catheter tips (*n* = 89)	32 (35.9%)	23 (71.9%)	14 (43.8%)	8 (57.1%)
Tracheal aspirates (*n* = 87)	42 (48.3%)	24 (57.1%)	21 (50.0%)	12 (57.1%)
Others^#^ (*n* = 145)	38 (26.2%)	13 (34.2%)	6 (15.8%)	3 (50.0%)
Hospital units				
OPD (*n* = 280)	118 (42.1%)	79 (66.9%)	41 (34.7%)	24 (58.5%)
General ward (*n* = 262)	64 (24.4%)	31 (48.4%)	27 (42.1%)	11 (40.7%)
ICU (*n* = 178)	98 (55.0%)	44 (44.9%)	20 (20.4%)	15 (75.0%)
Total (720)	280 (38.8%)	154 (55.1%)	88 (31.4%)	50 (56.8%)

*MDR* multi drug resistant, *ICU* intensive care unit, *OPD* out-patient department; others^#^ denotes throat swab, vaginal swab, synovial fluid, and pleural fluid, blood; *denotes percentage was calculated on growth positive, and **denotes percentage was calculated on *K. pneumoniae* isolates

### Antibiotic susceptibility testing

**Fig. 1:**
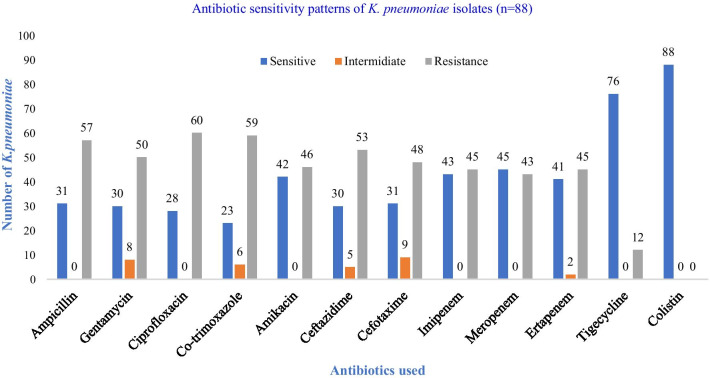
Antibiotic sensitivity patterns of *K. pneumoniae* isolates (*n* = 88)

Among the 12 different antibiotics used against *K. pneumoniae*, colistin was found to be the most effective (100% sensitive), followed by tigecycline (86.3% sensitive; 76/88), whereas co-trimoxazole was the least effective (26.1% sensitive; 23/88). Maximum sensitivity and resistivity to most of the antibiotics were found in *K. pneumoniae* isolated in urine and sputum sample, respectively (Additional file 1: Table S1). Among the carbapenems, meropenem was the most effective. A total of 65.9% (58/88) *K. pneumoniae* were resistant to at least one of the carbapenem antibiotics used and were labeled as suspected carbapenemase producers (Fig. 1).

### Phenotypic confirmation of carbapenemase-producing K. pneumoniae isolates

Out of 58 suspected carbapenemase producers, 45 (77.6%) were confirmed as such which constituted 51.1% (45/88) of the total *K. pneumoniae* isolates (Table 2). MHT positivity was higher in males than females. Among the samples, the highest percentage of carbapenemase-producing *K. pneumoniae* was obtained from the tracheal aspirates 71.4% (15/21), while among the wards, the highest prevalence was in the ICU 65.1% (13/20). The results were, however, statistically insignificant (*p* > 0.05).

**Table 2:** Distribution of MDR, MHT-positive and blaNDM-2 and blaOXA-48 genes-harboring *K. pneumoniae*

*K. pneumoniae* count (*N*)	Carbapenem resistant	MHT positive	p-value	blaOXA-48	blaNDM-2
	(%)*	(%)*		(%)**	(%)**
Sex					
Male (*n* = 37)	26 (70.3%)	23 (62.2%)	0.07	4 (17.4%)	6 (26.1%)
Female (*n* = 51)	32 (62.7%)	22 (43.1%)		3 (13.6%)	5 (22.7%)
Clinical samples					
Urine (*n* = 24)	13 (54.1%)	12 (50%)	0.367	2 (16.7%)	5 (41.7%)
Sputum (*n* = 23)	18 (78.2%)	10 (43.5%)		1 (10%)	2 (20.0%)
Catheter tips (*n* = 14)	9 (64.3%)	7 (50.0%)		1 (14.3%)	1 (14.3%)
Tracheal aspirates (*n* = 21)	16 (76.2%)	15 (71.4%)		2 (13.3%)	3 (20.0%)
Others (*n* = 6)	2 (33.3%)	1 (16.7%)		0 (0.0%)	0 (0.0%)
Hospital units					
OPD (*n* = 41)	24 (65.9%)	19 (46.3%)	0.627	3 (15.8%)	3 (15.8%)
General ward (*n* = 27)	16 (59.3%)	13 (48.1%)		1 (7.7%)	2 (15.4%)
ICU (*n* = 20)	18 (90.0%)	13 (65.1%)		3 (23.1%)	6 (46.2%)
Total	58 (65.9%)	45 (51.1%)		7 (15.6%)	11 (24.4%)

*Denotes percentages were calculated on *K. pneumoniae* count and **denotes percentages were calculated on MHT-positive *K. pneumoniae*

### PCR results

Out of 45 carbapenemase-producing isolates, 11 (24.4%) and 7 (15.5%) were found to be positive for blaNDM-2 and blaOXA-48 genes, respectively. The prevalence of these genes was higher in males. The highest percentages were found in urine samples (41.7%), and the sample of ICU patients, respectively. There was no significant association between the distribution of either gene among the clinical samples and the hospital units (Table 2).

## Discussion

The rapid spread of multidrug-resistant Gram-negative bacteria continues to be one of the most significant challenges to global health [27]. While these bacteria have developed a different mechanism to avert the bactericidal effects of commonly prescribed antibiotics, the increasing prevalence of carbapenemase-producing Gram-negative bacteria is of particular concern [28]. The rapid spread of mobile carbapenemase-containing genes, the limited treatment options, and the high mortality rates for infections associated with carbapenemase-producing isolates make them one of the major public health threats [8].

In this study, 38.8% of the samples were growth positive, with more than half were Gram-negative bacteria of which *K. pneumoniae* (31.4%) was the predominant isolate. This was in agreement with previous studies from Nepal [4]. Vibas et al. [29] reported a much higher, while Aryal et al. [30] reported a lower prevalence of *K. pneumoniae*. In this study, the highest percentage of *K. pneumoniae* was found in tracheal aspirates, followed by catheter tips. This was in contrast to that reported by Ferreiria et al. [31]. *K. pneumoniae* was isolated at the highest rate from the general ward, followed by the OPD and the least from the ICU. This was in contrast tothe study performed by Khan et al. [32]. The variation among the distribution *of K. pneumoniae* is considerable as the studies used for comparison are from different geographical locations with differing numbers of specimens used. Furthermore, the use of antibiotic therapy among patients, topographical situations, sanitation and the location of the study can also explain the variation in the distribution of *K. pneumoniae*.

Among the 12 different antibiotics used, colistin was 100% effective against *K. pneumoniae* while 86.3% isolates were sensitive to tigecycline. Thus, colistin and tigecycline could be the drug of choice for carbapenem-resistant *K. pneumoniae* isolates. Many researchers have also reported high sensitivity against colistin [2, 4, 30]. Ciprofloxacin showing maximum ineffectiveness was in agreement with previous finding in Nepal [19]. MDR is a global issue and a relatively high burden seen in developing countries [27]. Various prevalence of MDR *K. pneumoniae* have been frequently reported in Nepal [4, 17, 33]. In our study, more than half of isolates were MDR. High prevalence of MDR could be due to the easy availability and blind irrational use of antibiotics without proper culture report and prescription [34, 35].

Out of 88 K*. pneumoniae* isolates, half were MHT positive. Shanmugan et al. [36] reported a higher prevalence (82.6%) of MHT-positive *K. pneumoniae* in India*;* however, lower prevalence of carbapenemase-producing K. pneumoniae is also reported in India [14], and also in Nepal [37]. In this study, the highest percentage of carbapenemase was observed in tracheal aspirates (74.4%), followed by catheter tips. On the contrary, Rai et al. [38] reported the highest carbapenem-resistant *Klebsiella* isolates in urine followed by blood samples. In this study, almost two-thirds of the cases of carbapenemase production were seen in ICU patients and the lowest among OPD patients. Nair et al. also reported a similar trend of carbapenem-resistant isolates from the three different units of the hospital. This higher distribution of carbapenemase producers in the ICU may be related to excessive use of broad-spectrum antibiotics, associated septicemia, and higher comorbidities among ICU patients [39].

The prevalence of OXA-48 and NDM-2 among the MHT positive *K. pneumoniae* isolates in our study was 15.6% and 24.4%, respectively. Manandhar et al. [17] reported a similar prevalence of OXA-48, whereas Solgi et al. [40] reported a much higher prevalence than this study. In this study, the coexistence of both genes was found in 11.1% of the carbapenemase-producing isolates. Both the genes were predominantly found in isolates from urine. Huang et al. [41] reported the highest percentage of NDM gene in the blood and then followed by sputum. Among the wards, the isolation rates of both genes were high in ICU, which was in contrast with the findings of Gonzalez et al. [42]. The variations in the prevalence of the genes might be due to the difference in distribution pattern in different geographical regions and the pattern of antibiotics used.

The rapid increase of antibiotic-resistant genes like the OXA-48 and NDM-2 poses a significant threat to the modern world [28]. Irrational use of antibiotics, especially in developing countries like Nepal, further escalates the problem. Moreover, the low economy and the lack of well-trained human resources limit the use of genetic detection. In such cases, a relatively easy and inexpensive method like the MHT can be one of the best alternatives for the early detection of the carbapenemase producers [43] although it has several limitations like low specificity and high false positivity [8]. Incorporating of a needy but feasible molecular tests offers a reliable, cost-effective solution for the screening and preventing the dissemination of the MDR carbapenemase producers [9].

## Conclusions

A high prevalence of carbapenemase-producing *K. pneumoniae* was observed. The prevalence of blaOXA-48 and blaNDM-2 was 15.6% and 24.4%, respectively. Options for treating infections with carbapenemase-producing *K. pneumoniae* are limited, and colistin and tigecycline could be the drug of choice. In addition, routine surveillance of MBLs-producing bacteria is necessary to identify appropriate empirical antimicrobial therapy and restrain their spread in a hospital setting.

### Limitations of the study

The study was conducted in only one hospital using a small sample size which may not represent the whole population. Seasonal trend may also affect the result. The study was also limited to a single phenotypic confirmatory test and the detection of only two carbapenemase genes, although many other factors and genes are responsible for carbapenemase activity. Thus, the relationship shown among different factors in this study may not be conclusive. Thus, further studies should be performed in multiple hospitals for a longer period to overcome the present drawbacks.

## Supplementary Information

**Additional file 1: Table S1.** Samplewise antibiogram of *K. Pneumoniae* isolates.

## Data Availability

The datasets generated and analyzed during the current study are available in the supplementary materials repository.

## Abbreviations

AST: Antimicrobial susceptibility testing
MHT: Modified Hodge test
DNA: Deoxyribonucleic acid
PCR: Polymerase chain reaction
MDR: Multidrug-resistant
WHO: World Health Organization
NDM: New Delhi metallo-beta-lactamase
OXA: Oxacillinase
IRC: Institutional Review Committee
EDTA: Ethylenediamine tetraacetic acid
TAE: Tris–acetate-EDTA
